# New Absorbable Microvascular Anastomotic Devices Representing a Modified Sleeve Technique: Evaluation of Two Types of Source Material and Design

**DOI:** 10.1038/s41598-019-47499-5

**Published:** 2019-07-29

**Authors:** Woonhyeok Jeong, Kyuhee Kim, Daegu Son, Yunkun Kim

**Affiliations:** 1 Department of Plastic and Reconstructive Surgery, School of Medicine & Institute for Medical Science, Keimyung University Dongsan Medical Center, Daegu, Korea; 2 Metabiomed, Chungbuk, Korea

**Keywords:** Preclinical research, Translational research

## Abstract

The purpose of this study was to create a new absorbable vascular anastomotic coupler and evaluate the patency and degradation degree. Vascular anastomosis was performed in the jugular vein in 31 New Zealand white female rabbits. The coupler consisted of an inner and outer ring. One side of the jugular vein was passed through and overlapped the inner ring. The opposite side of the jugular vein overlapped the everted jugular vein on the inner ring. Then, the outer ring engaged with the inner ring and completed the anastomosis. The outer rings were also constructed with two shapes including an O-type that had no slit and a C-type with a slit on the outer ring of the O-type coupler to allows expandability of the diameter. A Phase I experiment was performed to evaluate the degradability of the source materials, including the poly (lactic-co-glycolic acid) (PLGA) and polycaprolactone (PCL) couplers. A Phase II experiment was performed to evaluate the patency and anastomosis time of the O-type PLGA and PCL couplers. A Phase III experiment was performed to evaluate the patency and anastomosis time of suture anastomosis (control) and the C-type PLGA coupler. The patency was determined by ultrasonography and open exploration. Histological analysis was performed to determine the degradability of the couplers. In Phase I, the PLGA couplers were completely degraded with good vascular wall remodeling at 8 months, while the PCL couplers demonstrated incomplete degradation. In Phases II and III, the anastomosis time was significantly shorter in the coupler groups than that in the control group. All of the coupler groups demonstrated complete patency of the anastomoses on ultrasonography. In Phase III, the C-type PLGA coupler also demonstrated patency and complete degradation at 8 months. PLGA is a suitable source material for absorbable couplers due to its fast degradability. We devised the O-shaped outer ring for the C-shaped outer ring to increase flexibility, which also demonstrated complete patency during the experimental period. Our absorbable microvascular anastomosis devices could provide rapid and reliable microvascular anastomosis without anastomotic failure.

## Introduction

After the omental flap and groin flap were successfully transferred using microvascular anastomoses in 1972 and 1973^1,2^, reconstructive microsurgery rapidly became increasingly popular and coincided with advances in surgical microscopes and microsurgical instruments. Although flap failure dramatically decreases with maturation of a surgeon’s skill level^3^, due to the learning curve associated with micro-anastomosis, anastomotic failure can still occur due to several causes, such as tearing, leaking, narrowing of the lumen, through-stitching, and inclusion of the adventitia^4^. Especially, veins are vulnerable to anastomotic failure because of their thin and fragile vascular wall. To overcome the difficulty of vein anastomosis, various types of vascular couplers have been developed for microvascular anastomosis without the use of sutures to improve vascular patency, as well as the ease and speed with which anastomoses are performed^5–8^.

The most frequently used anastomotic coupler in clinical practice is the MAC coupler^®^ system (Synovis Life Technology, Birmingham, AL, USA). The MAC coupler^®^ system is widely adapted to venous anastomosis and significantly reduces the operative time, decreases venous thrombosis, and improves flap survival compared with hand-sewn venous anastomosis techniques^9^. Indeed, the MAC coupler^®^ system is mostly used in venous anastomosis because the arteries have a thick wall compared to veins and cannot be easy everted to engage with a coupler system. It is composed of six metal pins inserted into a polyethylene ring at regular intervals. Compared to existing techniques using suture material, the MAC coupler has been reported to make the process of vascular anastomosis both easier and faster, with a higher rate of vascular patency^6^. However, it has the disadvantage that the ring and pins are not absorbed into the body. The remaining rings can press on peripheral vessels and lead to chronic inflammation or even extrusion over time when they are not covered with a thick skin flap^10,11^. Therefore, we sought to create an absorbable vascular anastomotic coupler that degrades quickly after vascular remodeling at an anastomotic site. Because a ring-pin system is difficult to create using absorbable materials, we invented a new coupler system design imitating the sleeve technique for microvascular anastomosis. The purpose of this study was to create a new absorbable vascular anastomotic coupler and evaluate the chronological change of degradation degree according to two types of material and the patency of two types of coupler design.

## Materials and Methods

### Absorbable microvascular anastomotic coupler designed for use with veins with a diameter of 2 to 3 mm

**Figure 1:**
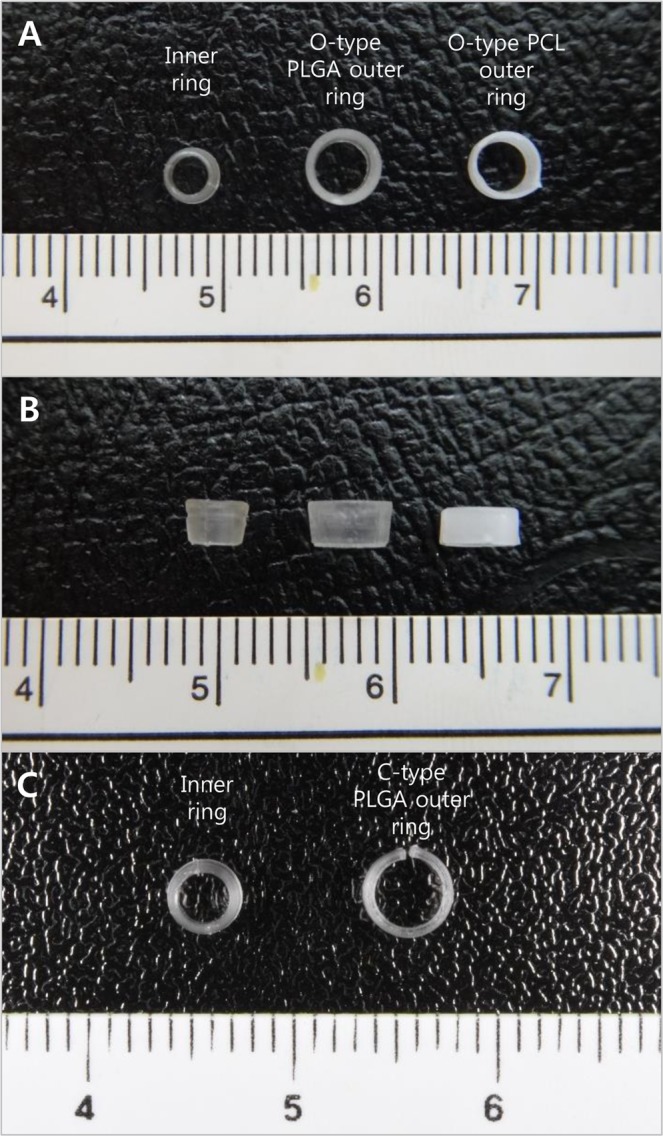
O-type PCL, O-type PLGA, and C-type PLGA couplers. (**A**) Axial view of the couplers. The inner ring of all couplers is composed of PLGA. The O-type PCL coupler consists of a PCL outer ring without a slit and a PLGA inner ring. The O-type PLGA coupler consists of a PLGA outer ring without a slit and a PLGA inner ring. (**B**) Sagittal view of the couplers. The inner ring has a tapering diameter that is narrowest at the end closest to the anastomotic site and increases toward the opposite side. (**C**) Axial view of the C-type PLGA coupler. The outer ring has a slit to facilitate union with the inner ring because PLGA exhibits stiff characteristics.

A new absorbable anastomotic coupler was developed and created through design, mold, and injection processes (Meta Biomed Co., Ltd., Cheongju, Chungbuk, Korea). The coupler consists of an inner ring and an outer ring. The inner ring is made of poly (lactic-co-glycolic) acid (PLGA), which is composed of 20% lactic acid and 80% glycolic acid. The ratio of lactic acid to glycolic acid was investigated, and the selected material was determined to be the most appropriate to create an absorbable vascular anastomotic device in terms of degradation time and strength^12^. The inner ring is composed of only PLGA because it does not need to be expandable during engagement with the outer ring. The outer ring is composed of either polycaprolactone (PCL) or PLGA. The two source materials of the outer rings were used to allow comparisons between the two substances with respect to the ease of anastomosis and the degree of absorption. The inner ring has a tapering diameter that is narrowest at the end closest to the anastomotic site and increases toward the opposite side. The inner ring has a groove that aligns with a ridge on the inside of the outer ring when the outer ring is inserted over the inner ring. The inside and outside diameters of the inner ring are 2.5 mm and 3.83 mm, respectively. The inside and outside diameters of the outer ring are 3.12 mm and 4.3 mm, respectively. The diameter of the inner ring was designed for application in veins 2 to 3 mm in diameter, which is the most commonly used diameter range in free flap surgery. The outer rings were also constructed as two shapes: O-type and C-type. The C-type outer ring has a slit that allows expandability of the diameter to facilitate union with the inner rings using PLGA (Fig. 1).

### Vascular anastomosis technique

Prior to beginning the study, the experimental research plan was approved by the Laboratory Animal Ethics Committee of Keimyung University School of Medicine (KM-2012-65R). All procedures in studies involving animals were performed in accordance with the Laboratory Animal Ethics Committee of Keimyung University School of Medicine’s guidelines and regulations. Thirty-one female New Zealand white rabbits (Orientbio, Seongnam, Gyeonggi, Korea) weighing approximately 4 kg were used.

**Figure 2:**
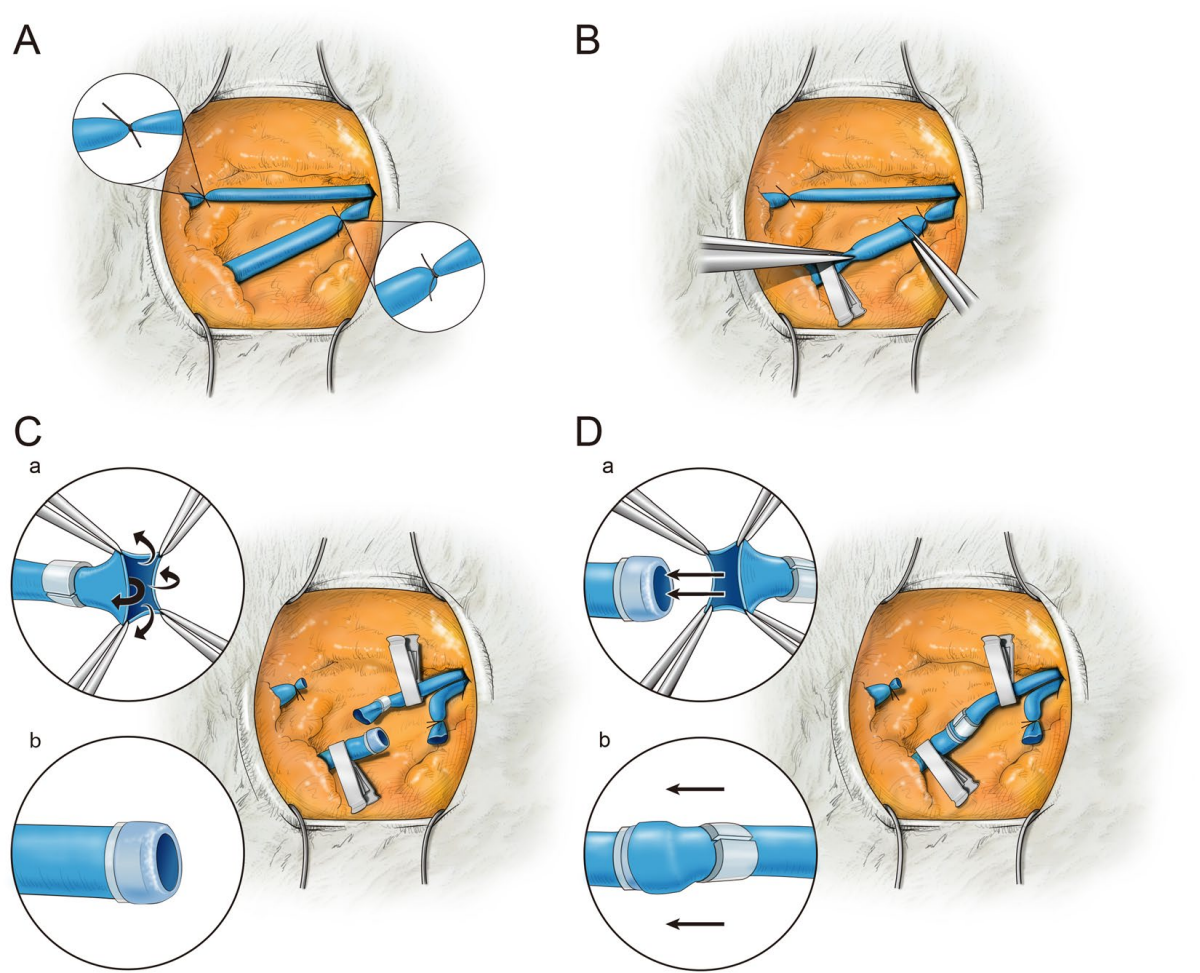
Modified sleeve technique for microvascular anastomosis. (**A**) The cephalad external jugular vein and the caudal internal jugular vein were ligated to acquire a sufficient length of the vessel. (**B**) The external and internal jugular veins were cut after applying vascular clamps. (**C**) The end of the external jugular vein was everted with jeweler’s forceps to form a cuff. (**D**) The internal jugular vein was passed through the outer ring. The internal jugular vein was positioned so that it overlapped the external jugular vein and the inner and outer rings were brought together.

Construction of the absorbable anastomotic coupler was based on the modified sleeve anastomosis technique. After endotracheal intubation, general anesthesia was maintained with inhaled isoflurane (Foran; Choongwae Pharmaceutical Corporation, Hwaseong, Gyeonggi, Korea). A longitudinal incision 7 cm long was made 2–3 cm lateral to the midline of the neck. The external and internal jugular veins were exposed carefully under a surgical microscope (Zeiss OPMI® Pico; Carl Zeiss, New York, NY, USA). All surgical procedures were recorded with an integrated camera coupled to the surgical microscope. The external jugular vein was anastomosed in transposition to the internal jugular vein to avoid the increased tension produced by shortening the total length of the vessel, which is a characteristic of the sleeve technique (Fig. 2A). The most cephalad portion of the external jugular vein and the most caudal portion of the internal jugular vein were anastomosed to obtain a sufficient vessel length. First, the external and internal jugular veins were separated from the surrounding soft tissues, followed by application of vascular clamps to the external and internal jugular veins (Fig. 2B). The external and internal jugular veins were cut, and their lumens were irrigated with heparin-containing normal saline. After sufficient adventectomy, the anastomosis end of the external jugular vein was pulled through the inside of the inner ring using jeweler’s forceps. The end of the external jugular vein was then everted and pulled over the inner ring (Fig. 2C). The caudal end of the corresponding internal jugular vein was passed through the outer ring and positioned over the everted external jugular segment to form a sleeve-type vascular anastomosis. While maintaining this vascular connection, anastomosis was completed by sliding the outer ring over the inner ring until the outer ring’s ridge was aligned with and fixed to the inner ring’s groove (Fig. 2D and Video 1). In the control group, vessel anastomosis was performed using a 9–0 nylon suture following the traditional technique of three stay sutures at 120 degrees; otherwise, the procedure was identical to anastomosis using a coupler. The experiment was performed in three phases.

### Experimental schedule

**Figure 3:**
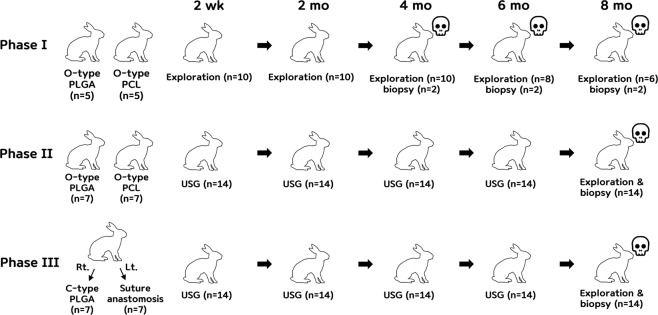
Schematics to explain the experimental schedule.

The Phase I experiment was performed as a preliminary experiment to determine the degradability of the O-type PCL and PLGA couplers and the chronological changes in vascular remodeling. The Phase II experiment was performed to determine the anastomosis time, vascular patency, and vascular remodeling at 8 months with the O-type PCL and PLGA couplers. In the Phase III experiment, we incorporated the O-type PLGA coupler into the C-type PLGA coupler for expandability because the outer ring of the O-type PLGA coupler has stiff characteristics and is not easy to expand during engagement with the inner ring. The Phase III experiment was performed according to the same experimental schedule using the O-type PLGA and C-type PLGA couplers (Fig. 3).

### Phase I: Preliminary evaluation of the degradability of the source material for a coupler in the process of time

This experiment was performed using an O-type coupler that did not have a slit in the outer ring. The experiments were performed in two groups of animals: the O-type PLGA group (5 rabbits and 5 anastomotic sites; n = 5), in which vascular anastomosis was performed with a PLGA outer ring/PLGA inner ring, and the O-type PCL group (5 rabbits, 5 anastomotic sites; n = 5), in which vascular anastomosis was performed with a PCL outer ring/PLGA inner ring. The vessels were anastomosed using the technique explained.

In the O-type PCL and PLGA groups, the degree of absorption of the coupler material was evaluated by open exploration through a previous operative scar in all rabbits at 2 weeks, 2 months, 4 months, 6 months, and 8 months after vascular anastomosis. The degree of absorption of the coupler material was evaluated by open exploration with photography. The degree of degradation was analyzed by whether it maintained or deformed the shape and maintained or slipped out of integration.

After general anesthesia was induced, one of 5 rabbits was euthanized by an intravenous injection of 20 mL of potassium chloride (KCl 2 mEq/mL, JW Pharmaceutical, Seoul, Korea) for biopsy at 4 months and 6 months, followed by biopsy of the remaining 3 rabbits at 8 months. Vessel remodeling, patency, absorption of the coupler material, inflammation, and other potential histological findings were evaluated by histological examination of biopsy specimens. A sufficient length of vessel that included the vascular anastomosis site was collected in each biopsy specimen. The collected vessel was washed and fixed in 10% neutral buffered formaldehyde for 1 week. The vessel was subsequently divided in the median plane (under microscope visualization) and embedded in paraffin to make 5-µm paraffin blocks. Slides were stained with hematoxylin and eosin (HE staining) and evaluated by optical microscopy.

### Phase II: Evaluation of anastomosis time and patency of the O-type couplers

The experimental group was divided into two groups: the O-type PCL group, which was composed of the PLGA inner ring/PCL outer ring (7 rabbits and 7 anastomotic sites; n = 7), and the O-type PLGA group, which was composed of the PLGA inner ring/PLGA outer ring (7 rabbits and 7 anastomotic sites; n = 7).

### Anastomosis time

Anastomosis time was counted in a recorded video of the surgical procedure. Vascular anastomosis time was defined as the time from completing the adventectomy by blocking vessel flow by applying the vascular clamp to remove the vascular clamp when completing anastomosis by using the absorbable anastomosis coupler. After anastomosis, the vascular clamp was removed and the anastomosis site was observed to determine whether there was any leakage. Vascular patency was evaluated immediately after surgery by the empty-and-refill test.

### Determining intravascular blood flow

Vascular patency was evaluated by ultrasonography at 2 weeks, 2 months, 4 months, and 6 months after vascular anastomosis. The rabbits were sedated by intramuscular injection of ketamine (Yuhan Ketamine 50^®^; 25 mg/kg, Yuhan Pharmaceutical, Seoul, Korea) for immobilization, and their neck hair was shaved. Lubricant was applied to the surgical area and duplex ultrasonography (SonoVet R5®; Samsung Medison Co., Ltd., Seoul, Korea) was performed in the sagittal and axial planes to evaluate the vessel’s patency and the presence of vessel leakage or clot formation. At 8 months, all rabbits underwent open exploration to determine the patency of the anastomoses, followed by biopsy to obtain specimens.

### Phase III: Evaluation of anastomosis time and patency of the C-type PLGA coupler and suture anastomosis

Because the O-type PLGA outer ring was integrated into the inner ring, the author experienced difficulty with insertion, and a minor crack was noted on the outer ring in two cases because PLGA was not resilient. To overcome this problem, the author modified the O-type PLGA coupler to a C-type PLGA coupler that had a slit on the outer ring to allow for widening of the diameter during integration.

In 7 rabbits, one side of the neck was treated using suture material (control group; n = 7) and the other side of the neck was treated using C-type PLGA couplers (C-type PLGA group; n = 7). Anastomosis time was also calculated on the recorded video in the C-type PLGA and control groups as performed in Phase II. Ultrasonography was performed at 2 weeks, 4 months, and 6 months as performed in Phase II. At 8 months, open exploration and biopsy were performed as in Phase II.

### Statistical analysis

The anastomosis time was analyzed by the Kruskal-Wallis test with Dunn’s post hoc test using GraphPad Prism 5® (GraphPad Software Inc., La Jolla, CA, USA) and is presented as the median value with the range. Values of p < 0.05 were considered statistically significant.

## Results

### Phase I: PLGA couplers were completely degraded with good vascular wall remodeling at 8 months, while PCL couplers demonstrated incomplete degradation

**Figure 4:**
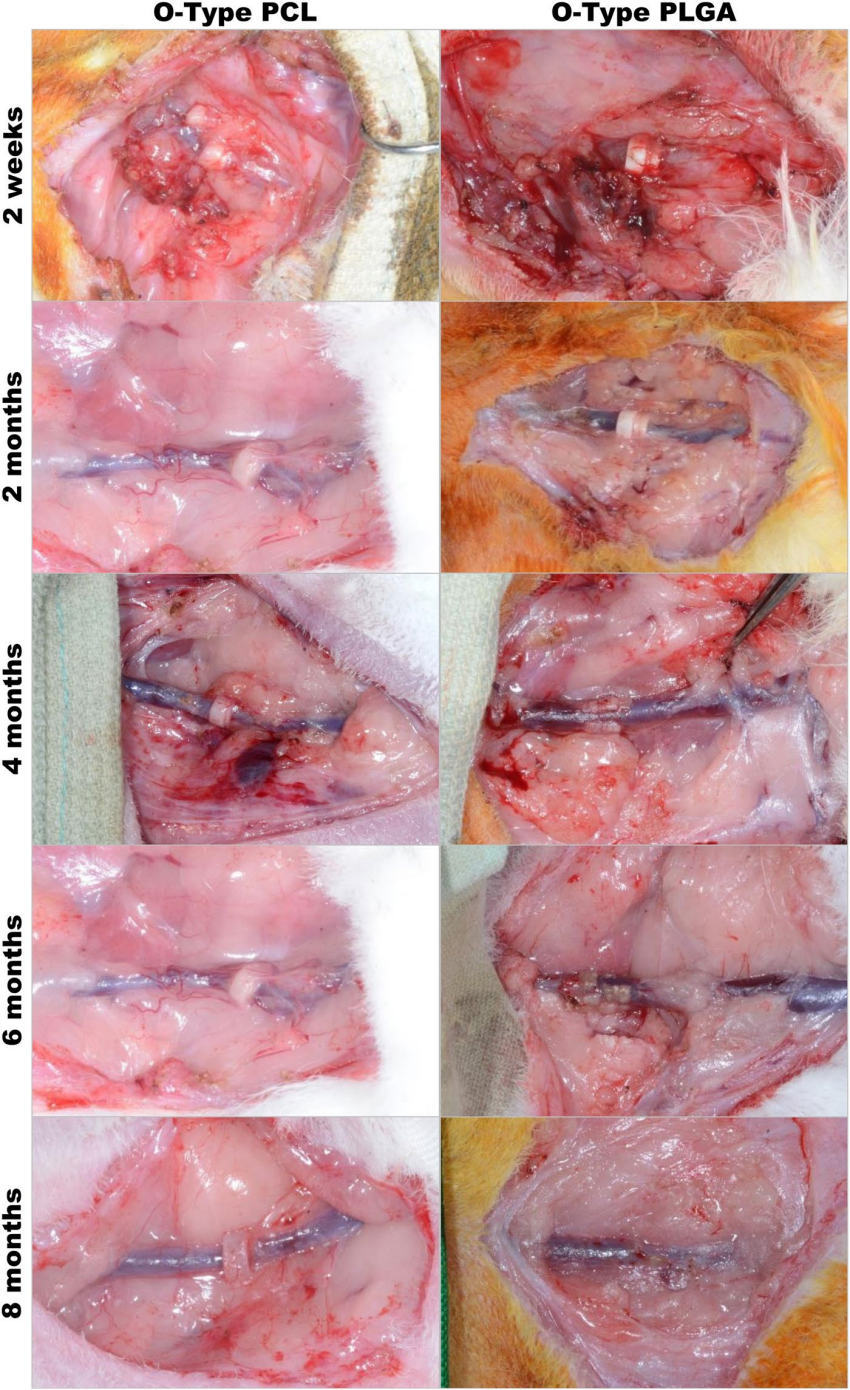
Degree of degradability of the O-type PCL and PLGA couplers. The O-type PCL group demonstrated grossly intact integration without degradation until 8 months. The O-type PLGA coupler exhibited an opaque white color and slightly slipped out of the outer ring at 2 weeks. At 4 months, the O-type PLGA coupler demonstrated marked degradation, followed by complete degradation with vascular remodeling at 8 months.

No clot formation or leakage was detected during microscope examination at 2 weeks or at 2, 4, 6, or 8 months after vascular anastomosis. In the O-type PCL group, the connective tissue was beginning to surround the vessel at 2 weeks after surgery. After 2, 4, 6, and 8 months, only one coupler demonstrated a slight slip-out of the outer ring. Every vascular anastomosis coupler was becoming smaller and thinner but its shape was maintained until 8 months (Fig. 4). Although all of the O-type PLGA couplers demonstrated intact integration without degradation at 2 weeks, all of the O-type PLGA couplers demonstrated degradation with significant deformity at 6 months and complete degradation at 8 months (Fig. 4).

**Figure 5:**
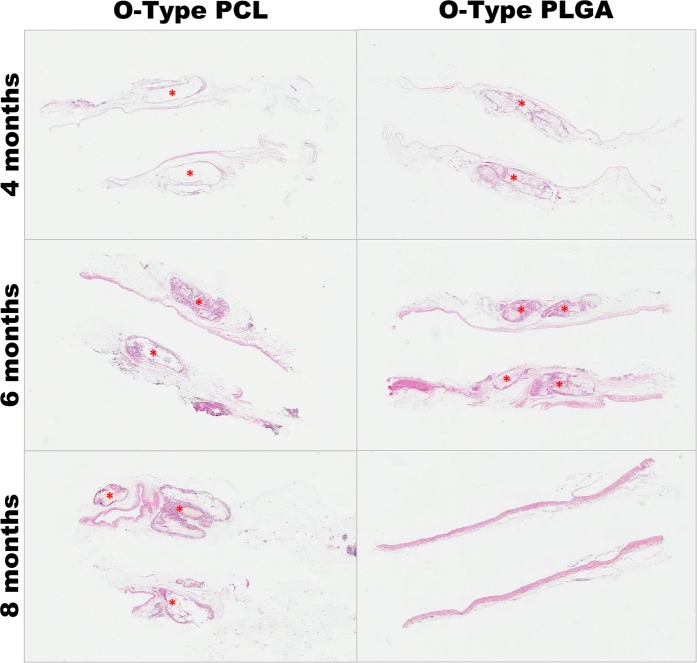
Histologic findings of the O-type PCL and PLGA couplers. Hematoxylin and eosin (HE) staining (×1) showed remodeling of the intimal layers in both the PCL and PLGA groups at 4 months. However, the O-type PLGA coupler demonstrated marked inflammatory cell infiltration into the coupler (red asterisk) with degradation. The O-type PCL group demonstrated significant inflammatory cell infiltration after 6 months. At 8 months, the O-type PLGA coupler was completely absorbed with remodeling of the whole vascular wall. However, the O-type PCL coupler exhibited marked inflammation at 8 months.

Based on HE staining, the O-type PCL and O-type PLGA couplers remained and were not degraded completely at 4 and 6 months. However, the O-type PLGA coupler exhibited slip-out with inflammatory cell infiltration into the coupler, while the O-type PCL coupler maintained integration with weak inflammatory cell infiltration at 4 months. At 6 months, the O-type PCL couplers also demonstrated inflammatory cell infiltration. At 8 months, the O-type PLGA couplers were completely degraded with good remodeling of the vessel wall. However, the O-type PCL couplers remained with ongoing degradation (Fig. 5). Based on these results, the author hypothesized that PLGA is a more suitable material for the outer ring because of its fast degradation after vascular wall remodeling. Thus, the author performed further experiments to evaluate this hypothesis.

### Phases II & III: Anastomosis time in the coupler groups was significantly decreased compared with that in suture anastomosis

The anastomosis times of the control, O-type PCL, O-type PLGA, and C-type PLGA groups were 26.3 (range, 19.3 to 32.5), 6.3 (range, 5.3 to 11.3), 7.3 (range, 5.0 to 12.0), and 8.5 (range, 5.3 to 12.5) min, respectively. The O-type PCL, O-type PLGA, and C-type PLGA groups demonstrated a significant decrease in anastomosis time compared to the control group (p < 0.001). When engaging the outer ring and inner ring of the O-type PLGA coupler, we experienced difficulty because PLGA has stiff characteristics. However, no significant difference was found among the coupler groups.

### Phases II & III: All of the O-type PCL, O-type PLGA, and C-type PLGA couplers demonstrated patency of the anastomoses until 8 months on ultrasonographic examination

**Figure 6:**
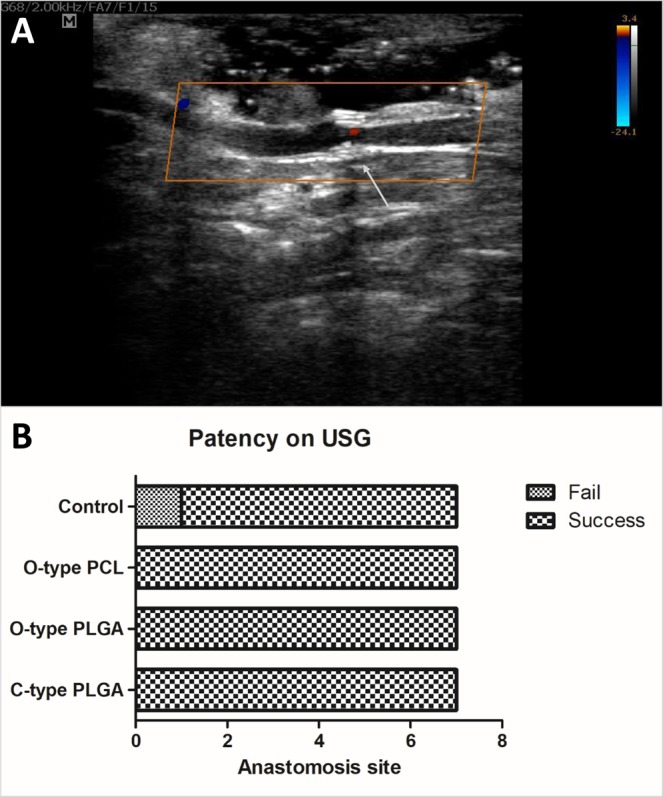
Ultrasonographic findings (USG) of anastomosed rabbit jugular veins. (**A**) The coupler is shown as hyperechoic echogenicity with posterior shadowing (arrow). Intraluminal flow was also detected by color duplex sonography. (**B**) Until 6 months, all coupler groups demonstrated intact intraluminal flow in ultrasonography. In one case in the control group, the author could not find an appropriate diameter of the jugular vein from 2 weeks, which was concluded to be a case of anastomotic failure.

Ultrasonographic examination was performed in the control, O-type PCL, O-type PLGA, and C-type PLGA groups at 2 weeks, 2, 4, and 6 months after vascular anastomosis. The anastomosis site of the coupler group revealed a perivascular hyperechoic lesion with intravascular flow in duplex scanning (Fig. 6A). None of the O-type PCL, O-type PLGA, or C-type PLGA couplers exhibited peripheral seroma, leakage, or clot formation at the vascular anastomosis site. However, one case in the control group demonstrated thrombosis and a reabsorbed jugular vein on ultrasonography at 2 weeks, which was concluded to be a case of anastomotic failure (Fig. 6B).

### Phases II & III: The O-type PLGA and C-type PLGA couplers demonstrated complete vascular remodeling on histology in contrast to the O-type PCL coupler that remained at 8 months

At 8 months, the author performed open exploration to determine the patency of the anastomoses and the degree of degradation and to perform a biopsy. The control group demonstrated complete vascular wall remodeling, except for one case in which the anastomosis thrombosed and the jugular vein was reabsorbed. This case demonstrated no flow on ultrasonography from 2 weeks. All of the O-type PCL couplers demonstrated a retained shape of the outer ring and patency of the anastomosis without gross degradation. Although inflammatory cells infiltrated into the couplers with degradation, the coupler was preserved as a round shape. In the O-type PLGA and C-type PLGA groups, all of the couplers were completely degraded grossly with patent anastomosis. Histological analysis revealed that the vascular wall was completely remodeled with weak inflammation in the perivascular area.

**Figure 7:**
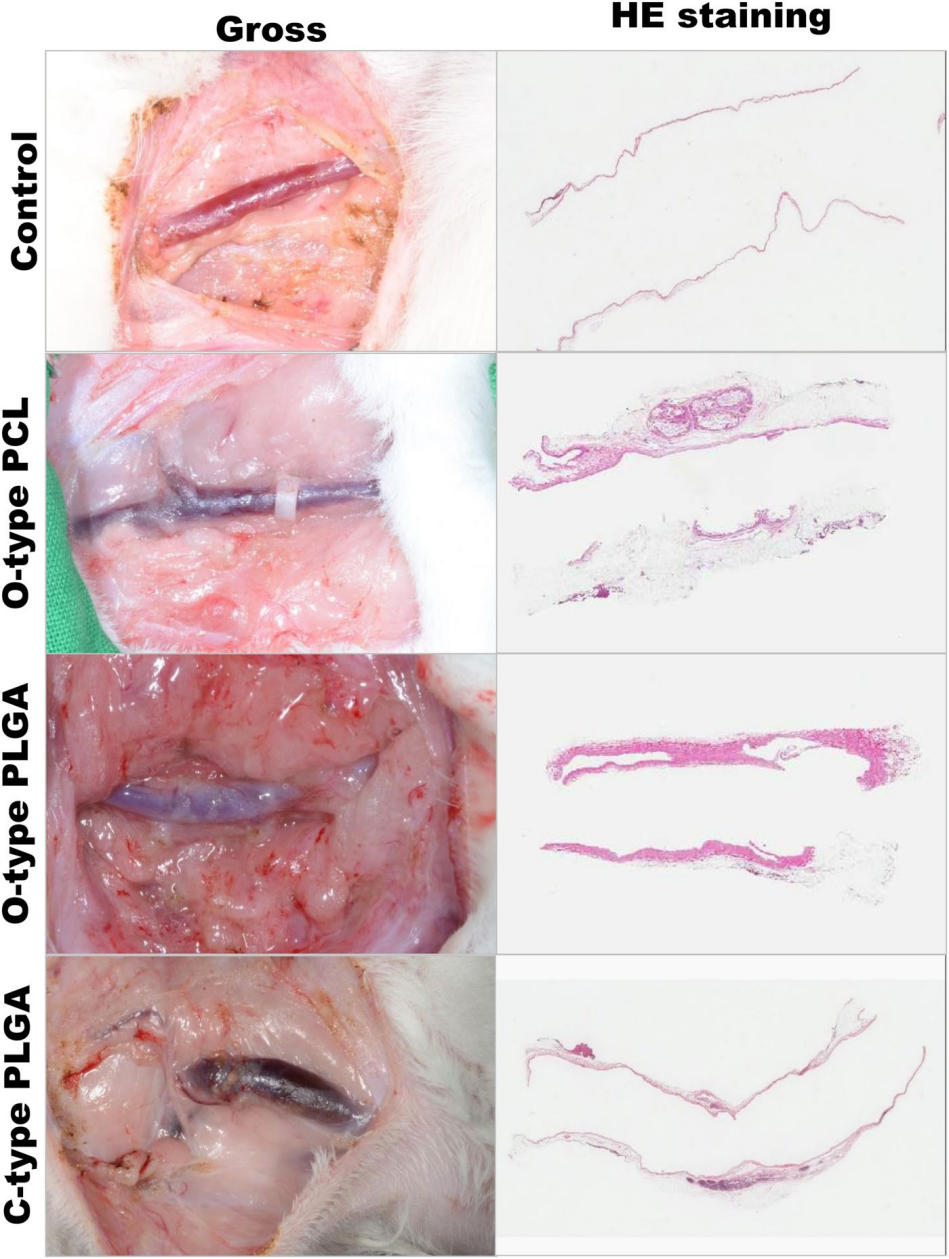
Gross and histologic findings at 8 months. The control group demonstrated complete remodeling of the vascular wall in gross and histological findings (HE staining; ×1). The O-type PCL group demonstrated no degradation and an intact outer ring in the gross findings. The inflammatory cells infiltrated into the PCL outer ring with intimal remodeling in the histological findings. The O-type and C-type PLGA couplers demonstrated complete degradation with vascular remodeling in the gross and histological findings.

After 4 months, histological examination of the anastomosed vessels in both groups revealed a completely regenerated intimal layer of vascular endothelial cells, but the medial and adventitial layers had not yet matured. Inflammation around the vascular anastomosis coupler was more active in the PLGA group than that in the PCL group but was limited to the area around the coupler, and inflammatory cells were not observed around the endothelial cells (Fig. 7).

## Discussion

In this study, the author invented a micro-anastomotic coupler based on PCL and PLGA source materials. The prototype of the coupler was composed of O-shaped outer and inner rings. The O-type PCL couplers were not degraded until 8 months, while the O-type PLGA couplers were fully degraded. The author modified the O-shaped outer ring of the O-type PLGA coupler to a C-shaped outer ring to overcome the difficulties of integration and minor cracks. The C- type PLGA coupler demonstrated complete degradation with preserved patency at 8 months.

The most frequently used vascular anastomosis device in current clinical practice, the MAC^®^ coupler, is based on a non-absorbable ring-pin system. Although anastomosis is accomplished faster and more easily than with manual suture techniques^11^, cases of the MAC^®^ coupler protruding have been reported because neither the polyethylene ring nor the stainless ring are absorbable^13^. To overcome this disadvantage, absorbable vascular anastomosis couplers have been developed. An absorbable ring with a stainless steel pin system mimics the MAC^®^ system^14^. However, an 11% failure rate caused by separation of the absorbable coupler after anastomosis has been reported. Another study reported that couplers containing hook-shaped pins increased the frictional force of the pins attaching to the ring to prevent separation of the couplers^15^. Although the study reported complete patency of this coupler, it was composed of an absorbable ring and non-absorbable stainless pins; therefore, it was not completely absorbable. The absorbable vascular anastomosis coupler described in the current study is an improvement over couplers used in previous systems because it is an interlocking system; it has a “coupler-on-coupler” in which the inner ring fits firmly into the outer ring to prevent separation by a wide contact surface. In this regard, all couplers maintained their integration without dislodging at 2 weeks in the Phase I experiment and demonstrated patency until 8 months in the Phase II and III experiments.

Although the coupler presented in this study reproduces the sleeve technique, the author modified the sleeve technique at the anastomotic site. The sleeve technique that was first introduced by Lauritzen^16^ is a variant of end-to-end anastomosis, where one side end of the vessel is placed into the other side end of the vessel similar to a telescope, i.e., “end-in-end” anastomosis. However, the sleeve technique was not popularized because of its low success rate^17^. Although the authors did not discuss the reason for the low success rate, potential causes may be separation of the anastomosis and clot formation in the overlapped vessels^16^. Furthermore, exposure of the adventitia to the lumen of the vessel may be another cause of thrombosis due to remnant adventitia of the inserted vessel. In the coupler presented in this study, the inserted vessel was everted through the inner ring, and eventually, and only the intima of the vessel was inserted into the opposite vessel. Accordingly, the possibility of exposure of the adventitia may be lower with this coupler system than with the conventional sleeve technique and even with end-to-end anastomosis because adventitia invagination occurred due to tight tying or incomplete eversion during suturing. Furthermore, intima-to-intima contact was important because such contact facilitated full remodeling of the tunica media and intima in an experiment involving an absorbable vein coupler^18^. Regarding this technical point of anastomosis using coupler systems, every vascular coupler device, all of which are commercially available, has been applied to venous anastomosis in clinical practice because the coupler system is not easy to apply to arterial anastomoses. Arteries have a thick wall compared to veins and cannot be easy everted to engage with a coupler system. Furthermore, most microvascular surgeons are less concerned about arterial anastomosis because arteries are technically easy to manage and anastomose. In fact, microvascular surgeons use a microvascular anastomotic device only for venous anastomosis, and published data on microvascular anastomotic devices apply only to venous anastomosis^9^.

The polymer components of the absorbable rings, PCL and PLGA, demonstrated excellent biocompatibility and safety in a hematologic *in vitro* study^19^. PCL is more flexible than PLGA, which leads to easier and potentially more accurate anastomosis, but it requires 36 months to degrade completely^20^. Accordingly, the O-type PCL coupler was not degraded until 8 months. PLGA is less flexible than PCL, but it has the advantage of being completely absorbed within 6–8 months^21^. In this study, inflammation around the coupler was most active at 4 months after anastomosis in both groups; therefore, one can infer that the components begin to be absorbed within 4 months after anastomosis. The PLGA group exhibited greater inflammatory cell infiltration around the coupler than the PCL group, indicating that more inflammation leading to earlier absorption occurred in the former group. Eventually, the PLGA polymer was completely absorbed by 8 months after anastomosis without evidence of vascular stricture. Over time, the PLGA vascular anastomosis coupler was associated with greater maturity of the intimal endothelial, medial, and adventitial layers than the PCL coupler. Therefore, the author concludes that PLGA is a suitable source material for absorbable microvascular anastomotic devices. When the O-type PLGA coupler was integrated, the author could not complete the integration smoothly because of the stiffness of PLGA. Furthermore, a minor crack frequently developed in the outer ring, although a total break or dislodge did not develop in any case. Therefore, the author devised an outer ring for the O-type PLGA coupler to facilitate integration by creating a slit on the outer ring. A slit on the outer ring provided resilience to overcome the problem of minor cracking during integration.

In free flap surgery, the anastomosis time is directly related with warm ischemia time or venous congestion. In the case of venous or artery first anastomosis, a faster venous anastomosis time reduces the warm ischemia time or venous congestion, respectively, which is related to a buildup of free radicals^4^. Regardless of which vessel is anatomized first, prolonged clamping time is related to flap complications^22^. However, a surgeon can reduce the clamping time by using a coupler. In this experiment, the mean anastomosis times with all of the couplers were significantly faster than those for suture anastomosis. The anastomosis times of the coupler groups were decreased by approximately 20 min compared with suture anastomosis. Indeed, ultrastructural damage of the flap developed early, from 2 hours to 4 hours after warm ischemia^23^. Furthermore, an irreversible change in the flaps, including partial flap necrosis, could be developed at 4 hours after warm ischemia in the experimental model^23,24^. Therefore, a decrease in anastomosis time as a result of decreasing flap complications could be beneficial and save procedure times, such as the tourniquet time, flap inset, and flap tailoring, performed in warm ischemia.

However, some limitations existed in this experiment. Because this study was only an animal experiment and involved a small number of rabbits, the vascular anastomosis time and vascular patency rate results cannot be considered definitive objective data. Another limitation is that the sleeve technique used with this coupler wastes pedicle length compared with end-to-end anastomoses because the inverted length of the inserted vessel and the overlapped length of the opposite vessel are wasted. Therefore, this coupler may not be suitable for use with short vascular pedicle flaps. Despite these limitations, the coupler presented in this study has the prominent advantages of not impinging upon peripheral tissues, reducing the risk of foreign body reactions and thus minimizing the risk of complications, such as chronic inflammation and coupler material exposure, and allowing the recovery of natural vessel elasticity. Furthermore, future studies should be conducted to extend the application of our couplers to arterial anastomosis by developing various diameters of couplers and devising a coupler design.

## Conclusions

Using a rabbit jugular vein experimental model, self-made fully absorbable anastomosis coupler created anastomosis easily and quickly and was associated with a 100% vascular patency rate. It facilitated normal maturation of all three vessel layers. The PLGA coupler was completely absorbed by 8 months after surgery. Therefore, this basic science study suggests that this absorbable anastomosis coupler is a safe and fast method for performing microvascular anastomoses. Further investigations are warranted to accumulate more data and to provide objective evidence of its clinical effectiveness in humans.

## Supplementary information

Video 1
